# Performance of pulse palpation compared to one‐lead ECG in atrial fibrillation screening

**DOI:** 10.1002/clc.23595

**Published:** 2021-03-16

**Authors:** Katrin Kemp Gudmundsdottir, Tove Fredriksson, Emma Svennberg, Faris Al‐Khalili, Leif Friberg, Henrike Häbel, Viveka Frykman, Johan Engdahl

**Affiliations:** ^1^ Karolinska Institutet Dept. of Clinical Sciences, Danderyd University Hospital Stockholm Sweden; ^2^ Department of Medicine, Huddinge Karolinska University Hospital Stockholm Sweden; ^3^ Karolinska Institutet, Division of Biostatistics Institute of Environmental Medicine Stockholm Sweden

**Keywords:** atrial fibrillation, pulse palpation, screening, single‐lead ECG, atrial fibrillation, pulse palpation, screening, single‐lead ECG

## Abstract

**Background:**

The 2020 European Society of Cardiology atrial fibrillation guidelines recommend opportunistic screening for atrial fibrillation by pulse taking or ECG rhythm strip in those aged over 65 years.

**Hypothesis:**

We aimed to compare the diagnostic accuracy of pulse palpation to ECG rhythm strip when screening for atrial fibrillation. A secondary aim was to investigate whether participants with palpitations were more likely to be diagnosed with new atrial fibrillation.

**Methods:**

The study population were 75/76 year old individuals that participated in the STROKESTOP II study, a Swedish screening study for atrial fibrillation. Pulse palpation of the radial pulse for 30 sec was performed by healthcare professionals and recorded as regular or irregular. Thereafter a 30‐sec single‐lead ECG was registered. Patients were asked also if they had a history of palpitations.

**Results:**

Of the 6159 participants included in the study, 461 (7.5%) had irregular pulse. Twenty‐two (4.8%) of those with irregular pulse were diagnosed with atrial fibrillation on single‐lead ECG rhythm strip. Among those with regular pulse, 6 (0.1%) cases of new atrial fibrillation were found. The sensitivity of the pulse palpation test was 78.6% and positive predictive value 4.8%. The proportion of newly diagnosed atrial fibrillation was not different between those with and without history of palpitations.

**Conclusion:**

Pulse palpation was inferior to single‐lead ECG when screening for atrial fibrillation. We therefore advocate the use of single‐lead ECG rather than pulse palpation when screening for atrial fibrillation. Palpitations did not predict atrial fibrillation.

## 1 INTRODUCTION

Atrial fibrillation (AF) is a well‐known risk factor for stroke. In high‐risk individuals, the risk of AF‐associated stroke can be decreased by two thirds using oral anticoagulation (OAC) treatment.^1^ Unfortunately, in approximately 10% of patients, AF is first diagnosed when stroke has already occurred.^2^ A considerable proportion of patients have no symptoms from AF, often referred to as silent AF, but stroke risk in connection with AF is not dependent on symptoms.^3^ The combination of accurate diagnostic tests and a highly effective treatment for stroke reduction gives rise to the possibility of screening for AF in those at high risk; indeed AF seems to fulfill most of the criteria for population screening according to the World Health Organization.^4^

The 2020 European Society of Cardiology AF guidelines give a class I level B recommendation for opportunistic screening for AF by pulse taking in those aged over 65 years.^5^ The recommendation for pulse palpation is partially based on a randomized controlled trial from the United Kingdom by Fitzmaurice et al. in 2007. They detected the same amount of new AF at lower incremental cost by opportunistic pulse palpation compared to systematic 12‐lead ECG screening.^6^ Pulse palpation has shown varying sensitivity and specificity,^7^ but it is unarguably a low‐cost test. In recent years, numerous new methods for detecting irregular pulse as well as recording single‐lead ECGs have emerged.^8^ The tests for detecting irregular pulse can be considered triage tests, and ECG confirmation is necessary for AF diagnosis. The single‐lead ECG rhythm strips on the other hand do not need to be confirmed by a 12‐lead ECG for AF diagnosis if they show 30 s of AF. The yield and feasibility of handheld single‐lead ECG recordings in population‐based AF screening has been evaluated previously,^9^ and it has been shown to be cost‐effective in both opportunistic and systematic AF screening.^10^, ^11^

Palpitations are the most common symptom in AF patients, reported to occur in 40% of patients in one study.^12^ Symptoms of palpitation are a major driver for patients to seek medical attention, and often the reason for further efforts to find undiagnosed AF. A meta‐analysis from 2015 showed that the presence or absence of symptoms in AF patients was of no consequence for the risk of stroke.^13^ Whether or not it is more common to find previously undiagnosed AF during screening in subjects with a history of palpitations than among those without is unknown.

The primary aim of this study was to compare the accuracy of pulse palpation with single‐lead ECG rhythm strip when screening for AF. The secondary aim was to compare the yield of AF among participants with and without history of palpitations.

## 2 METHODS

### 2.1 Study design and participants

This is a sub‐study to The STROKESTOP II study, a prospective cohort study using NT‐proBNP as a risk‐stratifying tool in a systematic AF screening program for purposes of stroke prevention. The design and baseline results of the STROKESTOP II study have been published.^14^, ^15^ In brief all 75/76 year olds in the Stockholm region were identified by Statistics Sweden by their 10‐digit personal identification number assigned to all citizens in Sweden and randomized 1:1 gender‐ and age‐based to be invited to participate in the STROKESTOP II study or to serve as control group. There were no inclusion criteria other than year of birth and residence in the Stockholm region and no exclusion criteria.

Included in this study were all participants from the STROKESTOP II study who had no history of known AF. In the case where no pulse palpation had been performed, or if a single‐lead ECG was deemed uninterpretable and no 12‐lead ECGs were recorded as back up, the participant was excluded from further analysis. Data was collected between April 2016 and February 2018.

### 2.2 Patient and public involvement

The research questions were developed by the study team when designing the STROKESTOP II study. The patients were not involved in designing the study, choice of outcomes or the recruitment of the study. All participants received oral and written information and signed informed consent documents.

### 2.3 Test methods

Participants were asked to self‐report their medical history with regard to prior thromboembolic risk factors according to the CHA_2_DS_2−_VASc score, and if they had a history of palpitation symptoms.

Pulse palpation of the radial pulse for 30 s was performed by trained healthcare professionals. Regular pulse was defined as regular in rhythm and force. The pattern of irregularity was not especially defined. Irregular pulse was considered to be a positive test and recorded as such in the eCRF form.

Following the pulse palpation, a 30 s, handheld single‐lead ECG recording with the Zenicor ECG device (Zenicor Medical Systems, Stockholm, Sweden) was obtained and evaluated by the same health care professionals. The ECG analysis with this single‐lead ECG used a computerized algorithm marking all ECGs as normal or abnormal including those with poor signal quality or possible AF. The algorithm has been extensively validated, showing a 100% sensitivity of the system in identifying AF on an individual level and a negative predictive value of 99.99%.^16^ If the algorithm classified a recording as sinus rhythm, a 12‐lead ECG was not deemed necessary. If the algorithm indicated AF or if the signal quality was too poor for interpretation, a 12‐lead ECG was obtained for rhythm confirmation and a cardiologist from the study team was contacted for interpretation. AF was defined as irregular rhythm with no organized or regular atrial activity on an ECG. For the single‐lead ECGs, a minimum duration of 30 sec was required for diagnosis. The results from the single‐lead ECG (and/or 12‐lead ECG when deemed necessary) was used as reference standard as this is the case for diagnosing AF according to the ESC AF‐guidelines.^5^

Palpitation symptoms were self‐reported as yes/no to the question “Do you have palpitations?” by patients in a questionnaire before the visit. There was no request for a specification of character, duration or temporal context of palpitation symptoms.

Post hoc, all single‐lead ECGs were scrutinized by three cardiologists to confirm whether sinus rhythm could be clearly identified or whether a 12‐lead ECG should have been taken at the time. A sensitivity analysis was performed including participants from whom a 12‐lead ECG had not been obtained and the results from 2 weeks of intermittent (4 times daily) single‐lead screening following the single‐time point visit were imputed. Participants that had not concluded the additional 2 weeks of screening were excluded from the sensitivity analysis.

### 2.4 Analysis

Based on previous studies on pulse palpation, for reliable sensitivity and specificity, we needed at least 4984 participants to achieve 80% power to show significant difference between the two screening tests.^7^

Categorical variables were expressed as numbers and percentages. The demographics of individuals with and without new AF were compared using the Pearson's chi‐squared test for categorical variables.

Diagnostic accuracy parameters of the screening test were derived from a 2 × 2 contingency table. We reported the number of true positives, true negatives, false positives and false negatives. We calculated sensitivity and specificity, accuracy, positive and negative predictive values, likelihood ratios as well as the post‐test predictive values. Diagnostic accuracy parameters were expressed as means with 95% confidence intervals. We conducted subgroup analyses on the accuracy of the screening test for symptomatic versus asymptomatic participants and compared their area under the curve.

McNemar's test for dependent proportions was used to test whether the pulse palpation test was equivalent to the handheld‐ECG test. For all statistical comparisons a *p*‐value <.05 was considered significant.

We reported the results of this study, according to the Standards for Reporting of Diagnostic Accuracy Studies (STARD) statement. All analyses were performed using STATA/MP 15.1.

### 2.5 Ethics

The study complies with the Declaration of Helsinki, and the protocol was approved by the regional ethics committee in Stockholm (DNR 2015/2079–31/1). Written informed consent was obtained from all participants in the screening program. ClinicalTrials.gov identifier: NCT02743416.

## 3 RESULTS

### 3.1 Participants and data analysis

**FIGURE 1:**
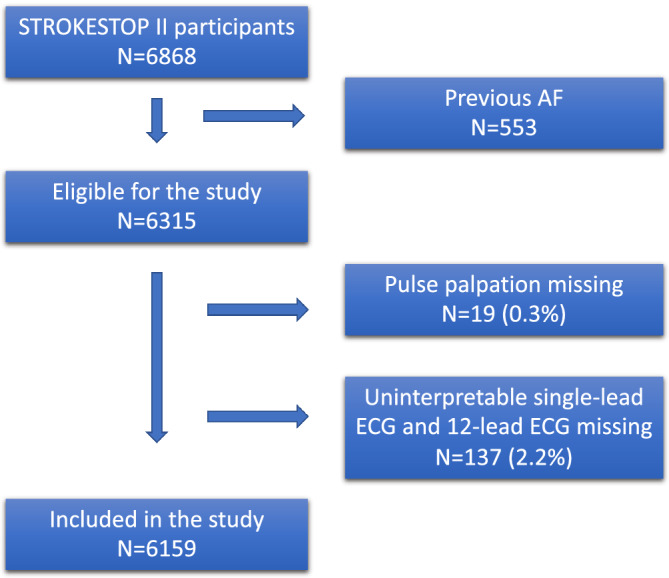
Study flow chart

In total, 6868 participants were included in the STROKESTOP II study. Previously known AF was found in 553 (8.1%) of the participants and those were excluded from analysis leaving 6315 participants eligible for this study. In addition, 19 (0.3%) participants had no pulse palpation performed and in 137 (2.2%) participants the single‐lead ECG was considered to be of insufficient signal quality with no 12‐lead ECG obtained, hence these were excluded, leaving 6159 participants, see Figure 1. Baseline characteristics of participants with regular and irregular pulse are shown in Table 1. Female sex, congestive heart failure, hypertension and history of palpitations were significantly more frequent in participants with irregular pulse.

**TABLE 1:** Baseline characteristics of participants with regular pulse and irregular pulse

Included participants	Regular pulse (*n* = 5698)	Irregular pulse (*n* = 461)	*p*‐value
Congestive heart failure *n* (%)	62 (1.1%)	11 (2.4%)	.013
Hypertension *n* (%)	2880 (50.5%)	259 (56.2%)	.020
Diabetes mellitus *n* (%)	614 (10.8%)	48 (10.4%)	.808
Stroke/TIA/thrombo‐embolism *n* (%)	401 (7.1%)	39 (8.5%)	.254
Vascular disease *n* (%)	349 (6.1%)	33 (7.2%)	.376
Female *n* (%)	3220 (56.5%)	215 (46.6%)	<.001
History of palpitations *n* (%)	1664 (29.2%)	163 (35.4%)	.005

### 3.2 AF screening accuracy of pulse palpation

**FIGURE 2:**
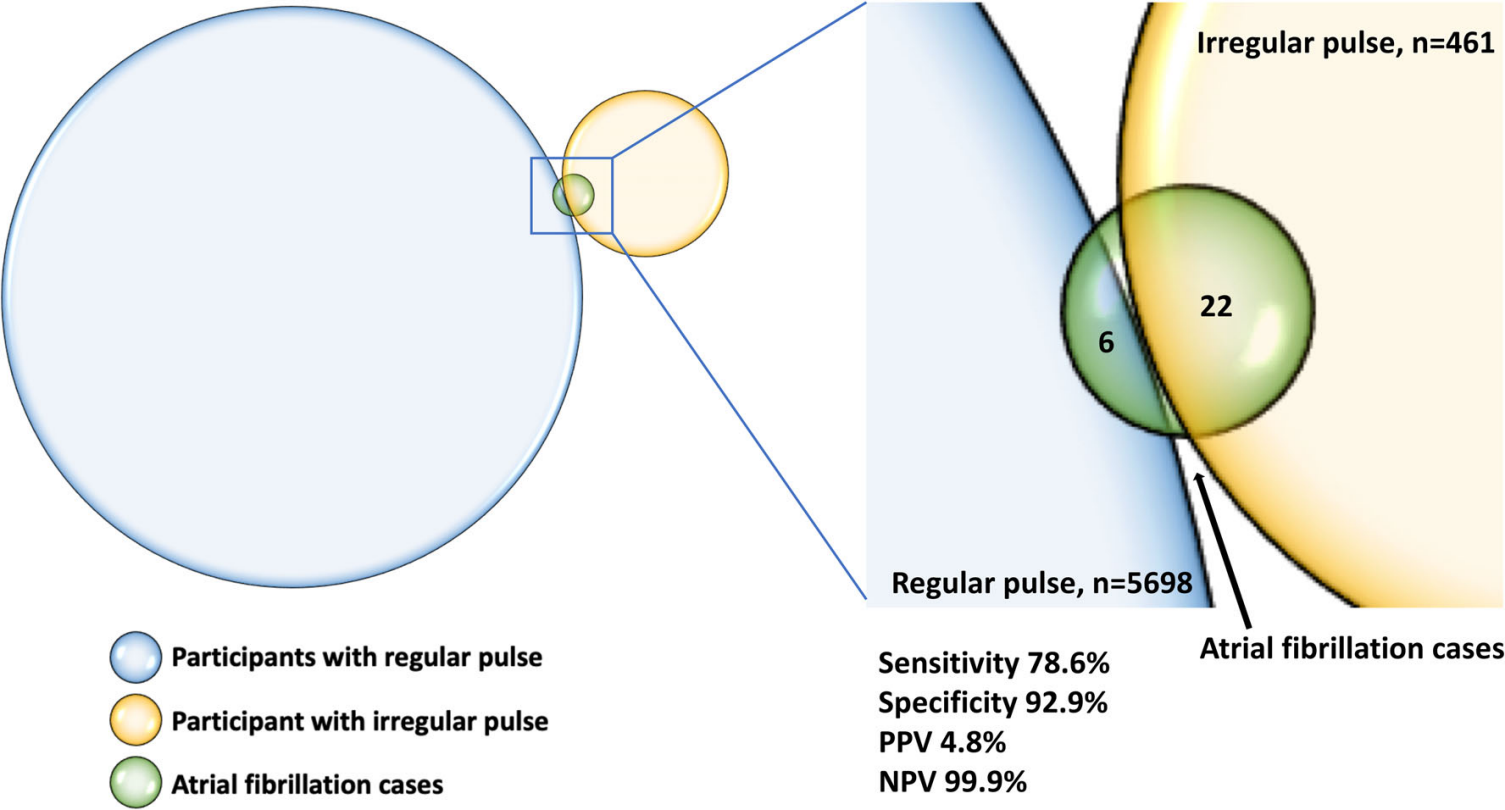
Diagnostic accuracy of pulse palpation

Of the 6159 participants, 461 (7.5%) had irregular pulse. Among these, 22 (4.8%) were diagnosed with AF. Among the remaining participants with regular pulse, *n* = 5698, 6 (0.1%) had AF on single‐lead ECG, *p* < .001. In total, new AF was diagnosed in 28 (0.5%) of all participants during the single time point screening visit. Sensitivity of pulse palpation was 78.6%, specificity 92.9%, positive predictive value 4.8% and negative predictive value 99.9%, yielding a total diagnostic accuracy of pulse palpation of 92.8%, see Figure 2. To diagnose one case of new AF after irregular pulse palpation, 21 ECGs had to be taken. No other significant brady‐ or tachycardia was identified on the single‐lead ECGs.

The test performance of pulse palpation compared to single‐lead ECG is shown in Table 2.

**TABLE 2:** Diagnostic performance of pulse palpation compared with one‐lead ECG

	Participants (*n* = 6159)
Irregular pulse, AF on single‐lead ECG (True positives)	22
Regular pulse, no AF on single‐lead ECG (True negatives)	5692
Irregular pulse, no AF on single‐lead ECG (False positives)	439
Regular pulse, AF on single‐lead ECG (False negatives)	6
Pre‐test probability (prevalence)	0.5% (0.3%, 0.7%)
Sensitivity (95% CI)	78.6% (59.0%, 91.7%)
Specificity (95% CI)	92.8% (92.2%, 93.5%)
Positive predictive value (95% CI)	4.8% (3%, 7.1%)
Negative predictive value (95% CI)	99.9% (99.8%, 100%)
Positive likelihood ratio (95% CI)	11.0 (8.7, 13.6)
Negative likelihood ratio (95% CI)	0.23 (0.1, 0.5)
Post‐test probability if positive likelihood ratio	5%
Post‐test probability if negative likelihood ratio	0%

Abbreviation: AF, atrial fibrillation.

A sensitivity analysis was performed where 93 of the 137 excluded participants missing a 12‐lead ECG had results imputed from 2 weeks of intermittent single‐lead screening following the single‐time point visit. The test performance of pulse palpation for the two different scenarios are shown in supplement Table 1.

### 3.3 Symptoms

There were 1827/6159 (29.7%) participants with history of palpitations. In symptomatic participants, new AF was diagnosed in 11 (0.6%). Most participants reported no history of palpitations, *n* = 4332 (70.3%) and AF was diagnosed in 14 (0.3%). There was no significant difference in the detection of new AF between symptomatic and asymptomatic participants. In participants diagnosed with new AF, 39% reported having had a history of palpitations.

The subgroup analysis for pulse palpation when stratified by history of palpitations is shown in supplement Table 2.

## 4 DISCUSSION

In this cohort study comprising over 6000 participants, pulse palpation showed a low positive predictive value and low sensitivity for AF detection when compared to a single‐lead ECG rhythm strip. For each positive pulse palpation test, more than 20 ECGs were needed to confirm one case of new AF. It is worth noting that about 30% of participants reported history of palpitations, but this did not predict new AF detection.

The European Society of Cardiology Atrial Fibrillation Guidelines from 2020 recommend pulse palpation in opportunistic screening for AF.^5^ Pulse palpation is a low‐cost and highly available, although non‐diagnostic test for AF, making verification with ECG necessary in case of irregular pulse. In our study, we found a lower sensitivity than that of Hobbs et al. found in the SAFE study resulting in a high false negative rate, regarded as an undesirable effect in a triage test.^17^ A study performed in a real‐life setting in general practice in Denmark, where a 12‐lead ECG was performed in case of irregular pulse, showed a similarly low positive predictive value for the pulse palpation test (5.1%) in the age group 75–84 years.^18^ In a multicenter primary care study in Sweden, where patients were instructed to palpate their own pulse and afterwards record a single‐lead ECG three times daily for 2 weeks, sensitivity was only 25% per measurement occasion.^19^ In the editorial on that paper, pulse palpation was called “unsuitable” in modern day, resource‐constrained health systems.^20^

Tests of irregular pulse are triage tests, whereas single‐lead ECG devices have shown good diagnostic accuracy.^9^, ^21^ A review from 2018 examined the evidence supporting the use of some of the newer technologies^8^ and in an era of rapid technological advances and the many emerging new instruments for detecting irregular pulse as well as easy to use single‐lead ECG recordings, it seems that there might be room for more modern and precise methods than pulse palpation.

The proportion of participants with new AF who reported history of palpitations (39%) was quite high, and similar to what Siontis et al (2016) found (40%) in their study on patients with previously known AF.^12^ We were expecting a lower share of symptomatic participants, as palpitations might have driven them to seek medical attention earlier, possibly detecting AF. In clinical practice, patients reporting symptoms of palpitations are assessed by pulse palpation as a triage test, but our results show that the post‐test probabilities of pulse palpation do not change with symptoms. Hence, history of palpitations did not predict new AF detection in our study.

Improved AF detection in high‐risk individuals has the potential of reducing the burden of AF‐related stroke and with many new techniques developing, accuracy studies are of essence. Important topics to consider are health outcomes, cost effectiveness, acceptability to the patients and ethical considerations when deciding supremacy of a new test. A single‐lead ECG takes about the same amount of time to perform as pulse palpation, but the diagnosis can be confirmed simultaneously. Pulse palpation might be a low‐cost triage test, but it must be followed by an ECG to confirm diagnosis. When a triage test has such a low positive predictive value, the number of false positives becomes considerable, the healthcare burden rises, and the gain of the test is canceled out with the cost of the second one. Ethically, pulse palpation as well as the single‐lead ECG, being non‐invasive and causing no physical pain for the patient, seem to be equal. A false positive pulse palpation test can cause considerable worries for the patient, especially if an ECG for confirmation cannot be performed without delay. If an ECG cannot be obtained simultaneously, it could leave room for doubt as to whether the heart rhythm was irregular at the time point of irregular pulse palpation. As for the health outcome, the negative effects of failure to recognize AF and thereby increasing the patient's risk for stroke cannot be stressed enough.

### 4.1 Study limitations

Measures of diagnostic accuracy are sensitive to study designs. A 12‐lead ECG is considered gold‐standard in rhythm diagnostics. Nonetheless, a single‐lead ECG showing a 30‐s AF is considered sufficient for the diagnosis of AF and the aim of this study was to diagnose either sinus rhythm or AF and not go into detailed rhythm interpretation. Single‐lead ECGs can be difficult to interpret if the signal quality is poor which necessitates additional examination with a 12‐lead ECG. In our study, a small proportion of single‐lead ECGs of poor quality went without a 12‐lead ECG confirmation and were excluded from the analysis. To try to compensate for this a sensitivity analysis was performed, including 94 out of 137 participants missing a 12‐lead ECG, with results from 56 single‐lead ECG registrations following the index visit imputed instead. This did not improve the performance of pulse palpation, in fact most accuracy parameters worsened in sensitivity analysis.

Pulse palpation was performed by trained healthcare professionals who had received detailed instructions and it is possible that this increased the accuracy of pulse palpation above what can be expected in general practice and by laymen. Furthermore, the health care professionals of the study staff performed large volumes of pulse palpations daily, which could have improved their skills further. There might also be observer variability as the subjective element cannot be excluded from pulse palpation. Although care was taken to ensure that pulse palpitation was performed without results of the single‐lead ECG, there is a possibility of review bias after the single‐lead ECG or clinical review bias as the patients reported their medical history. Observer variability between the study team cardiologist that confirmed single‐lead and 12‐lead ECG diagnosis is also possible.

The prevalence of the condition affects the performance of the test. Our study population was elderly, which means that both previously known AF as well as undiagnosed AF could be anticipated to have relatively high prevalence. Predictive values from this study should be carefully considered before applying the test results in different settings where the AF prevalence differ.

The temporal context of palpitations and timing of screening is an important question but unfortunately the participants were asked to answer a simple yes/no question and were not asked to specify any further, making it only possible to infer on history of palpitations.

## 5 CONCLUSIONS

Pulse palpation was inferior to single‐lead ECG when screening for atrial fibrillation. We therefore advocate the use of single‐lead ECG rather than pulse palpation when screening for atrial fibrillation. History of palpitations did not predict screening outcome.

## CONFLICT OF INTEREST

Dr. Katrin Kemp Gudmundsdottir has received lecture fee from Pfizer. Dr. Tove Fredriksson has received research grants from Boehringer‐Ingelheim, Stiftelsen Hjärtat and Capio Forskningsstiftelse. Dr. Emma Svennberg has received lecture fees from Bayer, Bristol‐Myers Squibb‐Pfizer, Merck‐Sharp & Dome, Boehringer‐Ingelheim, and Sanofi, as well as research grants from Boehringer‐Ingelheim. Dr. Johan Engdahl has received consultancy or lecture fees from Pfizer, Medtronic, Merck Sharpe & Dome and Bristol‐Myers Squibb. Dr. Faris Al‐Khalili has received lecture fees from Bristol‐Myers‐Squibb, Boehringer‐Ingelheim, and Bayer. Dr. Leif Friberg received consultancy fees from Sanofi, Bristol‐Myers‐Squibb, Bayer, Boehringer‐Ingelheim and Pfizer. Dr. Viveka Frykman reports lecture fees from Medtronic, MSD and Bayer. Henrike Häbel has no disclosures.

## AUTHOR CONTRIBUTION

Katrin Kemp Gudmundsdottir, Tove Fredriksson, Emma Svennberg, Faris Al‐Khalili, Leif Friberg, Viveka Frykman and Johan Engdahl conceived, planned and contributed all in the execution of the study. Katrin Kemp Gudmundsdottir, Emma Svennberg and Johan Engdahl were responsible for the rhythm decisions on single‐lead ECGs and 12‐lead ECGs. Henrike Häbel and Katrin Kemp Gudmundsdottir did the statistical analysis. Katrin Kemp Gudmundsdottir took the lead in writing the manuscript. All authors provided critical feedback and helped shape the research, analysis and manuscript. Katrin Kemp Gudmundsdottir is responsible for the overall content as guarantor.

## Supporting information


**Supplementary table 1** Sensitivity analysis

**Supplementary table 2** Diagnostic performance of pulse palpation compared to single‐lead ECG stratified by history of palpitations.

## Data Availability

The authors take responsibility for all aspects of the reliability and freedom from bias of the data presented and their discussed interpretation. The data that support the findings of this study are available from the corresponding author upon reasonable request.
