# Supplementary material for: Performance of pulse palpation compared to one‐lead ECG in atrial fibrillation screening
Source: Clin Cardiol. 2021 Mar 16;44(5):692–8. doi: 10.1002/clc.23595 (PMC8119837; doi:10.1002/clc.23595)
Supplement: Supplementary file 1 — Supplementary table 1 Sensitivity analysis [file CLC-44-692-s001.docx]

## Supplement table 1 Sensitivity analysis

|  | “Worst-case scenario” *  (n=6252) | “Best-case scenario” **  (n=6252) |
| --- | --- | --- |
| Irregular pulse, AF on single-lead ECG (True positives) | 24 | 22 |
| Regular pulse, no AF on single-lead ECG (True negatives) | 5772 | 5777 |
| Irregular pulse, no AF on single-lead ECG (False positives) | 445 | 447 |
| Regular pulse, AF on single-lead ECG (False negatives) | 11 | 6 |
| Pre-test probability (prevalence) | 0.6% (0.4%, 0.8%) | 0.4% (0.3%, 0.6%) |
| Sensitivity (95% CI) | 68.6% (50.7%, 83.1%) | 78.6% (59%, 91.7%) |
| Specificity (95% CI) | 92.8% (92.2%, 93.5%) | 92.8% (92.1%, 93.4%) |
| Positive predictive value (95% CI) | 5.1% (3.3%, 7.5%) | 4.7% (3.0%, 7.0%) |
| Negative predictive value (95% CI) | 99.8% (99.7%, 99.9%) | 99.9% (99.8%, 100%) |
| Positive likelihood ratio (95% CI) | 9.6 (7.5, 12.2) | 10.9 (8.8, 13.5) |
| Negative likelihood ratio (95% CI) | 0.34 (0.2, 0.6) | 0.2 (0.1, 0.5) |
| Post-test probability if positive likelihood ratio | 5% | 5% |
| Post-test probability if negative likelihood ratio | 0% | 0% |

Caption: A “worst-case scenario” was defined as if 7 of the participants had shown AF on index visit as was found during the following 2 weeks of screening. A “best-case scenario” was defined as if all 94 participants had shown sinus rhythm.

* 6159 participants + 7 imputed as AF + 87 imputed without AF

**6159 participants + 94 imputed without AF
