# Supplementary material for: Performance of pulse palpation compared to one‐lead ECG in atrial fibrillation screening
Source: Clin Cardiol. 2021 Mar 16;44(5):692–8. doi: 10.1002/clc.23595 (PMC8119837; doi:10.1002/clc.23595)
Supplement: Supplementary file 2 — Supplementary table 2 Diagnostic performance of pulse palpation compared to single‐lead ECG stratified by history of palpitations. [file CLC-44-692-s002.docx]

### Supplement table 2. Diagnostic performance of pulse palpation compared to single-lead ECG stratified by history of palpitations.

|  | History of palpitations (n=1827) | No history of palpitations (n=4332) |
| --- | --- | --- |
| Irregular pulse, AF on single-lead ECG (True positives) | 8 | 14 |
| Regular pulse, no AF on single-lead ECG (True negatives) | 1661 | 4031 |
| Irregular pulse, no AF on single-lead ECG (False positives) | 155 | 284 |
| Regular pulse, AF on single-lead ECG (False negatives) | 3 | 3 |
| Pre-test probability (prevalence) | 0.6% (0.3%, 1.1%) | 0.4% (0.2%, 0.6%) |
| Sensitivity (95% CI) | 72.7% (39.0%, 94.0%) | 82.4% (56.6%, 96.2%) |
| Specificity (95% CI) | 91.5% (90.1%, 92.7%) | 93.4% (92.6%, 94.1%) |
| Positive predictive value (95% CI) | 4.9% (2.1%, 9.4%) | 4.7% (2.6%, 7.8%) |
| Negative predictive value (95% CI) | 99.8% (99.5%, 100%) | 99.9% (99.8%, 100%) |
| Positive likelihood ratio (95% CI) | 8.5 (5.8, 12.6) | 12.5 (9.8, 16) |
| Negative likelihood ratio (95% CI) | 0.3 (0.1, 0.8) | 0.2 (0.1, 0.5) |
| Post-test probability if LR+ | 5% | 5% |
| Post-test probability if LR- | 0% | 0% |
